# Proteomic Analysis of Peri-Wounding Tissue Expressions in Extracorporeal Shock Wave Enhanced Diabetic Wound Healing in a Streptozotocin-Induced Diabetes Model

**DOI:** 10.3390/ijms21155445

**Published:** 2020-07-30

**Authors:** Rong-Fu Chen, Ming-Yu Yang, Ching-Jen Wang, Chun-Ting Wang, Yur-Ren Kuo

**Affiliations:** 1 Division of Plastic Surgery, Department of Surgery, Kaohsiung Medical University Hospital, Kaohsiung 807, Taiwan.; dr.chenrf@gmail.com (R.-F.C.); chuntingb20120@gmail.com (C.-T.W.); 2 Graduate Institute of Clinical Medical Sciences, College of Medicine, Chang Gung University, Tao-Yuan 333, Taiwan; yangmy@gmail.com; 3 Department of Otolaryngology, Kaohsiung Chang Gung Memorial Hospital and Chang Gung University College of Medicine, Kaohsiung 833, Taiwan; 4 Department of Orthopaedics, Kaohsiung Chang Gung Memorial Hospital, Kaohsiung 833, Taiwan; cjwang1211@gmail.com; 5 Faculty of Medicine, Regeneration Medicine and Cell Therapy Research Center, College of Medicine, Kaohsiung Medical University, Kaohsiung 807, Taiwan; 6 Department of Biological Sciences, National Sun Yat-Sen University, Kaohsiung 804, Taiwan; 7 Academic Clinical Programme for Musculoskeletal Sciences, Duke-NUS Graduate Medical School, Singapore 169857, Singapore

**Keywords:** proteomics, shock wave, diabetic wound healing, hemopexin, serpin

## Abstract

Our former studies have demonstrated that extracorporeal shock wave therapy (ESWT) could enhance diabetic wound healing but the bio-mechanisms remain elusive. This study investigated the changes of topical peri-wounding tissue expressions after ESWT in a rodent streptozotocin-induced diabetic wounding model by using the proteomic analysis and elucidated the molecular mechanism. Diabetic rats receiving ESWT, normal control, and diabetic rats receiving no therapy were analyzed. The spots of interest in proteome analysis were subjected to mass spectrometry to elucidate the peptide mass fingerprints. Protein expression was validated using immunohistochemical staining and related expression of genes were analyzed using real-time RT-PCR. The proteomic data showed a significantly higher abundance of hemopexin at day 3 of therapy but down-regulation at day 10 as compared to diabetic control. In contrast, the level of serine proteinase inhibitor (serpin) A3N expression was significantly decreased at day 3 therapy but expression was upregulated at day 10. Using real-time RT-PCR revealed that serpin-related EGFR-MAPK pathway was involved in ESWT enhanced diabetic wound healing. In summary, proteome analyses demonstrated the expression change of hemopexin and serpin with related MAPK signaling involved in ESWT-enhanced diabetic wound healing. Modulation of hemopexin and serpin related pathways are good strategies to promote wound healing.

## 1. Introduction

The non-healing wound ulcer is a major morbidity in diabetic patients which is usually accompanied by infection, inflammation, or even amputations. Various treatment modalities for diabetic foot ulcers have been applied but the results remain controversial [1]. The general principles of wound management including debridement, infection control, endovascular intervention, or bypass surgery for ischemic ulcers, and hyperbaric oxygen therapy, topical wound dressings, etc., remain the recommended procedures [1,2]. Studies have shown that tissue ischemia induced by leukocyte mediated inflammation and insufficient neovascularization suppressing epithelization are the main factors that predispose a patient to poor wound healing and could result in mortality in patients with diabetes [3].

Extracorporeal shock wave therapy (ESWT) has been applied in clinical treatment for patients with urinary stones to disintegrate urolithiasis, tendinitis, or plantar fasciitis, epicondylitis of the elbow, etc. [4,5]. The biological mechanisms of ESWT are generally proposed to enhance tissue regeneration by angiogenesis enhancement [6,7]. Our former studies demonstrated topical ESWT accelerates skin flap tissue survival [6,8] and increases chronic wound healing [9,10]. Our results showed the main mechanism of ESWT significantly increased angiogenesis and topical blood perfusion, and enhanced cell proliferation and suppression of topical tissue inflammatory response [9].

Proteomic technology is a well-established tool for detecting the differential expression of various proteins in liquid biopsy, cell lines, and particularly in pathological tissue. In our previously utilized proteomic technology to illustrate the changes in systemic serum protein expressions in chronic wound healing after ESWT in a rodent diabetic wounding animal study. Our study revealed that numerous specific proteins were involved in ESWT enhancing wound healing such as haptoglobin, vitamin D-binding protein, etc. [11]. However, the application of proteomic tools to detect changes in topical wound edge tissue expression in chronic wound healing after ESWT has not been evaluated. Therefore, in this study, we further examined the changes of topical peri-wounding skin tissue expressions and detected the protein expressions of ESWT in the enhancement of the wound healing process.

## 2. Results

### 2.1. Analysis of Two-Dimensional Electrophoresis Profiles

**Figure 1:**
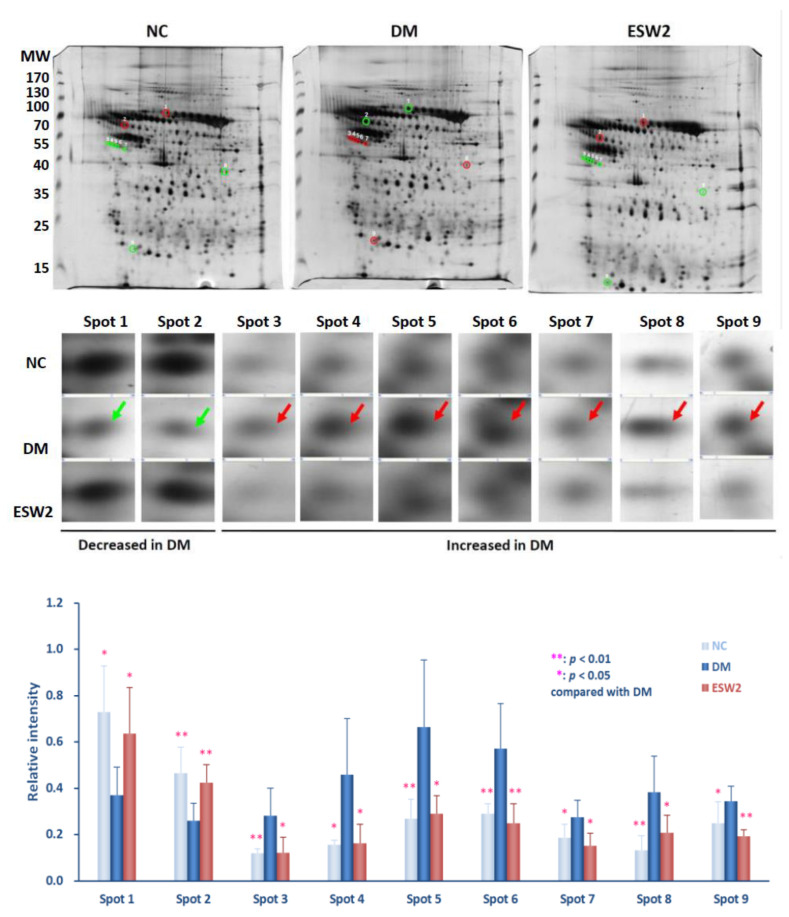
Protein spots of taking into consideration in 2D gel electrophoretograms of peri-wounding skin tissue proteins acquired from and a diabetic rodent on day 3 after two sessions of ESWT (**ESW2**), and that long period of normal control (**NC**) and diabetic control (**DM**). (**Above**) Representative 2D gel electrophoretograms of peri-wounding skin tissue proteins. The protein samples (150 µg) were exposed to isoelectric focusing (pH 4 to 7), SDS-PAGE, and silver staining. The numbers on the left demonstrate the molecular weight (*M*_W_), in kilodaltons. (**Center**) Enlarged fields of the 9 spots of interest in the sliver-stained SDS-PAGE gels. The spots in the gels of NC, DM, and ESW2 were coordinated utilizing Bio-Rad Proteoweaver 2-D Analysis Software Version 4.0. The arrows prevail upon the spots of interest. (**Below**) Relative densities of the emphatically distinguished proteins. Relative intensity was determined by separating the thickness of coordinated spots by the thickness of all the coordinated spots in the individual gel. Abbreviation: ESWT, extracorporeal shock wave therapy.

**Figure 2:**
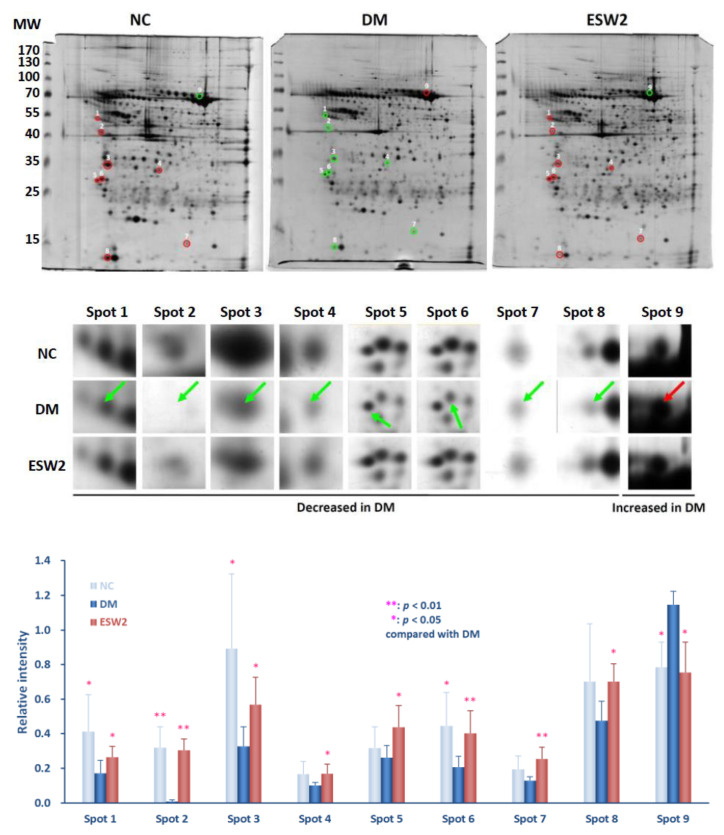
Protein spots of taking into consideration in 2D gel electrophoretograms of peri-wounding skin tissue proteins acquired from day 10 after two sessions of ESWT (**ESW2**), normal controls (**NC**), a diabetes controls (**DM**) at the same days. (**Above**) Representative 2D gel electrophoretograms of peri-wounding skin tissue proteins. The skin tissue protein samples (150 µg) were exposed to isoelectric focusing (pH 4 to 7), SDS-PAGE, and silver staining. The numbers on the left demonstrate the molecular weight (MW), in kilodaltons. (**Center**) Enlarged fields of the 9 spots of interest in the sliver-stained SDS-PAGE gels. The spots in the gels of NC, DM, and ESW2 were coordinated utilizing Bio-Rad Proteoweaver 2-D Analysis Software Version 4.0. The arrows prevail upon the spots of interest. (**Below**) Relative densities of the emphatically distinguished proteins. Relative intensity was determined by separating the thickness of coordinated spots by the thickness of all the coordinated spots in the individual gel. Abbreviation: ESWT, extracorporeal shock wave therapy.

Two-dimensional electrophoresis was assessed using peri-wounding skin tissue samples received from animals in the normal control group, diabetic group, and the group that received ESWT at days 3 and 10 after treatment. Results showed that approximately 218 spots (218.2 ± 36.5 spots in the normal control group, 215.6 ± 13.1 spots in the diabetic control group, and 219.8 ± 43.4 spots in the ESWT group) (*p* > 0.8) could be found in the gels (Figure 1 and Figure 2). Protein spots in each rat of the ESWT group were further imaged and matched with those in the normal group and diabetic control group, and the relative thickness of individual spot in each matched gel was computed. On day 3 after ESWT, the densities of nine protein spots were significantly distinct, as shown in Figure 1. Two of the nine spot intensities were increased and seven were decreased in ESWT rats, as compared to that in diabetic controls (Figure 1). In contrast, the intensities of nine protein spots were the significant difference on day 10 post-ESWT (Figure 2). All of the intensities of nine protein spots were increased in ESWT except one which was decreased, as reflated with the diabetic controls (Figure 2).

### 2.2. Protein Analysis and Identification

To illustrate the peptide mass fingerprints, the protein spots of taking into consideration (days 3 and 10 after ESWT) were exposed to in-gel trypsin digestion and matrix-assisted laser desorption ionization time-of-flight (MALDI-TOF) mass spectrometry. The peptide mass information of each spot was submitted to the National Center for Biotechnology Information and SWISS-PORT bioinformation stations utilizing Mascot web search tools. Nine spots of appropriately were truly identified as hemopexin (Spot 1), alpha-2-HS-glycoprotein (Spot 2), serine protease inhibitor A3N (serpin A3N, Spots 3, 4, 5, 6, and 7), leukocyte elastase inhibitor A (Spot 8), and catechol O-methyltransferase (Spot 9) at day 3 after ESWT treatment, as contrasted and those in controls. At day 10 after ESWT treatment, the nine spots of taking note of were completely identified as serpin A3N (Spot 1), tektin-4 (Spot 2), tropomyosin alpha-1 chain (Spots 3), plectin-1 (Spot 4), lamin-A (Spot 5), tropomyosin alpha-4 chain (Spot 6), ferritin heavy chain (Spot 7), myosin regulatory light chain 2, skeletal muscle isoform (Spot 8), and hemopexin (Spot 9). The mass spectrometric attributes of the distinguished proteins are summarized in Table 1 and Table 2.

The outcomes uncovered ESWT bunch had fundamentally more elevated levels of hemopexin (*p* < 0.05) and alpha-2-HS-glycoprotein (*p* < 0.01) and essentially lower levels of serpin A3N (*p* < 0.01), leukocyte elastase inhibitor A (*p* < 0.05), and catechol O-methyltransferase (*p* < 0.01) at day 3 after ESWT treatment, when contrasted with the diabetic controls (Figure 1, below). At day 10 after ESWT treatment, the outcome uncovered a noteworthy upregulation of serpin A3N (*p* < 0.05), tektin-4 (*p* < 0.01), tropomyosin alpha-1 chain (*p* < 0.05), plectin-1 (*p* < 0.05), lamin-A (*p* < 0.05), tropomyosin alpha-4 chain (*p* < 0.01), ferritin heavy chain (*p* < 0.05), and myosin regulatory light chain 2, skeletal muscle isoform (*p* < 0.05) and downregulation of hemopexin (*p* < 0.05) when contrasted with that in diabetic controls (Figure 2, below).

### 2.3. Detection of Hemopexin and Serpin A3N Expression Using Immunohistochemical Staining

**Figure 3:**
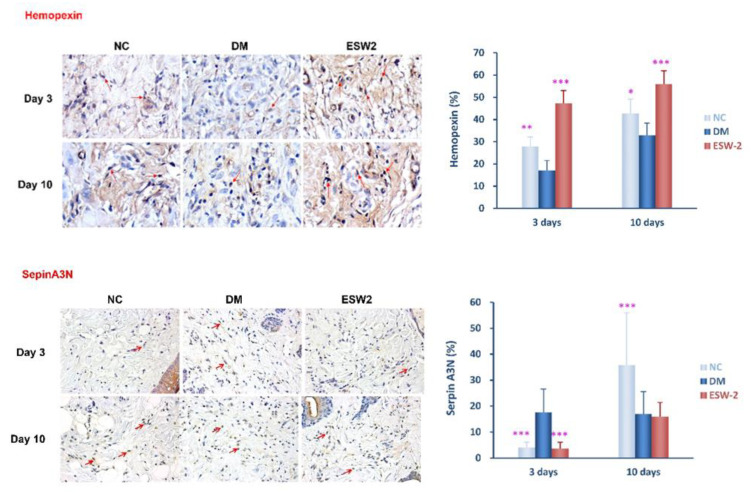
Hemopexin and serine protease inhibitor (SERPIN) A3N expression of peri-wounding tissue samples were analyzed by using immunohistochemical staining at day 3 among extracorporeal shock wave therapy (ESWT), normal controls (NC), and diabetic control (DM) groups. The original magnification is 400× and representative microscopic fields are shown. (**Above**) Cells positively stained for hemopexin antibody are also shown. *** *p* < 0.001. (**Below**) Cells positively stained for serine protease inhibitor A3N antibody are also shown. * *p* < 0.05. ** *p* < 0.005. *** *p* < 0.001.

Since the proteome analysis revealed the changes of specific proteins after ESWT, we further confirmed protein expression by IHC staining. As shown in Figure 3, the IHC staining assessment displayed that hemopexin expression was statistically increased and serpin A3N expression was statistically decreased in the ESWT group at day 3 after treatment when contrasted with that in diabetic controls (*p* < 0.001). These outcomes were steady with our finding observed in 2D gel electrophoresis of peri-wounding tissue samples.

### 2.4. ESWT-Enhanced Wound Healing Is Associated with Early Activation of EGFR Pathway

The study showed serpin as an epithelial barrier involved in epidermal growth factor receptor (EGFR) activation [12]. Cell proliferation and angiogenesis are important roles via regulating the EGFR-MAPK pathway [13,14]. We recognized EGFR-MAPK signal cascade related genes’ mRNA levels of a peri-wounding tissue test by quantitative RT-PCR rather than traditional Western blotting at the beginning time (three days) and late-stage (10 days) after ESWT.

**Figure 4:**
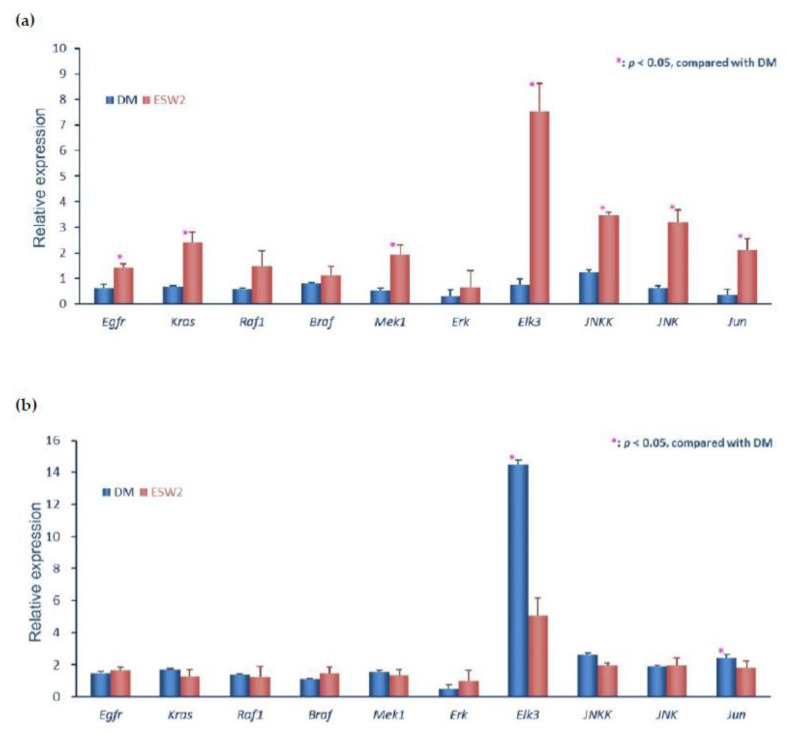
ESWT enhanced wound healing is associated with early activation of the epidermal growth factor receptor (EGFR) pathway. The expression levels of the genes of the EGFR signaling cascade by real-time quantitative RT-PCR were performed on mRNA samples extracted from biopsy test acquired from the transitional zone of the wound edge at day 3 (**a**) and day 10 (**b**) after two sessions of ESWT (ESW2), normal control (NC), control diabetic (DM) rats.

The results showed higher gene levels of Egfr, Kras, Mek1, Elk3, Jun, Jnk, and Jnkk at the beginning time (three days) in the ESWT group, when contrasted with that in diabetic rats (Figure 4a). However, the upregulation of Kras, Mek1, Elk3, Jnkk, Jnk, and Jun was strongly suppressed at the late stage (10 days) in ESWT rats, particularly the expression of Elk3 was reversed as compared to that in diabetic rats (Figure 4b). These results indicated EGF initiation of the Raf-MEK-ERK signaling is engaged with ESWT enhanced diabetic wound healing.

## 3. Discussion

Studies have indicated that ESWT speaks to a doable strategy for enhancing wound healing related to expanded epithelization and neo-angiogenesis, tissue recovery, and topical mitigating reaction [8,15]. In our former study utilized proteomic technology to distinguish the progressions in foundational serum protein profiling in diabetic wound healing after ESWT. Our results revealed that numerous proteins were involved in ESWT-treatment enhanced diabetic wound healing such as increased haptoglobin protein levels and downregulation of serpin A3N and vitamin D-binding protein on day 3 after treatment [11]. In this study, we extended our study and examine the protein expressions of topical per-wounding tissue in these diabetic rats using proteomic study and MALDI-TOF mass spectrometry.

The results revealed ESWT had significantly higher levels of hemopexin and alpha-2-HS-glycoprotein as well as significantly lower levels of serpin A3N, leukocyte elastase inhibitor A, and catechol O-methyltransferase at day 3 after treatment. On day 10 after ESWT treatment, noteworthy upregulated serpin A3N, tektin-4, tropomyosin alpha-1 chain, plectin-1, lamin-A, tropomyosin alpha-4 chain, ferritin heavy chain, and myosin regulatory light chain 2, skeletal muscle isoform and downregulated hemopexin was observed.

Recent studies have indicated that hemopexin and alpha-2-HS-glycoprotein had cytoprotective action against oxidative damage in cells [16]. The expression of skin tissue hemopexin was up- and downregulated, respectively, at three and 10 days after ESWT. In contrast, the expression of skin tissue serpin A3N was down- and upregulated, respectively, at three and 10 days after ESWT treatment. IHC analysis also revealed consistent results of hemopexin and serpin A3N expressions after ESWT. These indicated that hemopexin and serpin A3N both are involved in ESWT-accelerated diabetic wound healing.

Hemopexin is a heme-restricting plasma glycoprotein which, after haptoglobin, shapes the second line of barrier against hemoglobin-mediated oxidative injury [17]. Haptoglobin is the essential Hb-restricting protein in human plasma. Hemopexin is another plasma glycoprotein ready to tie heme with high partiality. Hemopexin and haptoglobin prevent heme’s pro-inflammatory and pro-oxidant effects and enhance its detoxification. Results of tissue proteomic analysis revealed significantly higher levels of hemopexin three days after ESWT and verified by IHC staining. This result is consistent with our former study that the serum of proteomic analysis in the ESWT-treated group showed upregulated haptoglobin protein levels [11]. These indicate ESWT modulation of hemopexin expression is involved in an earlier anti-inflammatory response and the suppression of oxidative stress to accelerate diabetic wound healing.

In addition to hemopexin, differential expressions of several proteins were identified in peri-wounding tissues that likewise assume a significant job in wound healing. Serpin A3N is an inhibitor of a few proteases, for example, cathepsin G, elastase, and chymase, made from neutrophils and mast cells, etc. [18]. Serpin A3 seems to have a multifaceted job and is related to inflammatory responses [19]. A study showed serpin A3N advances granulation tissue development and collagen deposition using a diabetic murine model [20]. In this study, our results revealed the down-regulatory effect of serpin A3N found in the peri-wounding tissue of the ESWT-treated group on day 3 but up-regulated at day 10 after ESWT. This might be serpin A3N related inflammatory response was suppressed in the early phase (day 3) after ESWT, but upregulation due to involved in enhancing the angiogenesis and tissue regeneration at later phase (day 10) after ESWT, as compared to the diabetic controls. These indicated modulation of serpin A3N expression could be an important factor in ESWT promotion of the wound healing process.

**Figure 5:**
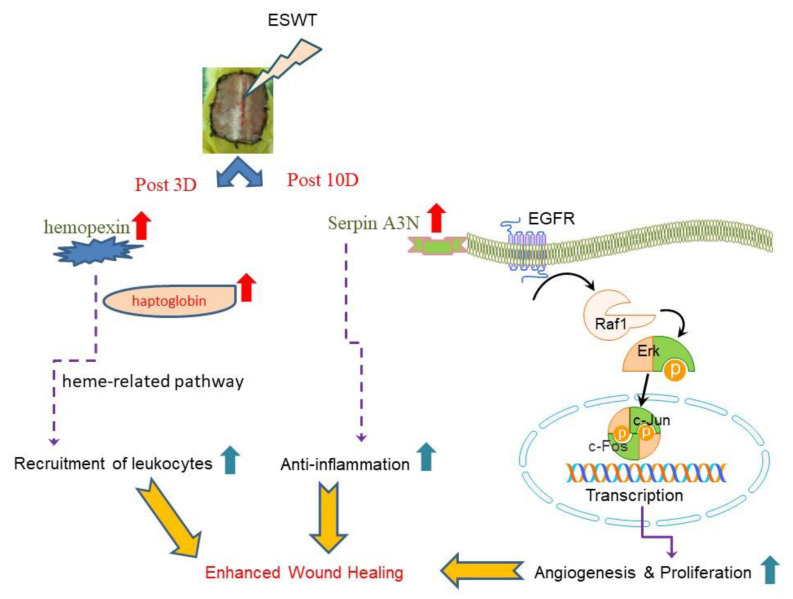
The purposed bio-mechanisms of ESWT improve wound healing are through modulation of hemopexin and Serpin with EGFR-MAPK pathways to increase cell proliferation and anti-inflammatory response. Abbreviation: ESWT, extracorporeal shock wave therapy; EGFR, epidermal growth factor receptor; Erk, extracellular-signal-regulated kinase; Serpin, serine protease inhibitor.

Studies showed the serpin involved in EGFR activation and epithelial barrier [12,21]. EGFR is an important role in angiogenesis and cell proliferation via regulating the Raf-MEK-ERK signaling [13,14]. In this investigation, real-time quantitative RT-PCR was performed on mRNA extracted from biopsy samples. Our results revealed that ESWT treatment could increase epithelial cell migration and cell multiplication and correlated with serpin-EGF initiates the Raf-MEK-ERK cascades to enhance diabetic wound healing (Figure 5).

Other factors have also been detected in this tissue proteomic study. Leukocyte elastase inhibitor A also called serpin B1A is an individual from the clade B of serpins [22]. It is an intracellular protein and acts fundamentally to shield the cell from proteases released into the cytoplasm during oxidative stress. Ongoing information demonstrates that it has likewise a job in cell migration proposing that it could be involved in dissimilar processes such as malignant metastases and wound healing [22]. Our results revealed that wounded diabetic rats that received ESWT had significantly lower levels of serpin A3N and serpin B1A on day 3 after treatment. More research has uncovered that serpins work in inflammatory response and infection, by regulating serine and cysteine proteases activities [23]. It might be due to the anti-inflammatory effects of ESWT, peri-wounding tissue samples had lower levels of serpin A3N and leukocyte elastase inhibitor A. Besides inflammation and proliferation, connective tissue regeneration is also an important process in wound healing. Tropomyosin α-1 chain and tropomyosin α-4 chain showed an increased expression in ESWT peri-wounding tissue samples at day 10 after treatment. It is noteworthy that tropomyosin is the archetypal-coiled coil, yet investigations of its structure and capacity have demonstrated it to be a dynamic controller of actin filament function in non-muscle and muscle cells [24]. This is an important finding in the understanding of the tropomyosin may be involved in the angiogenesis even remodeling in the enhancing diabetic wound healing by ESWT. Together, the present findings suggest that serpin and tropomyosin needed further study in detail.

In summary, our proteomic study of topical wound edge protein expressions reveals ESWT improving chronic wound healing involves in neo-angiogenesis and anti-inflammation. The modulation of hemopexin and serpin-related pathways will be a good strategy to promote wound healing (Figure 5).

## 4. Materials and Methods

### 4.1. Animal Investigations

The consideration and lodging states of the animals conformed to the guidelines of the Institutional Animal Care and Use Committee on the assurance of animals utilized for scientific purposes (IACUCA Animal use protocol approval number: 2007111902).

### 4.2. Streptozotocin (STZ)-Induced Diabetes Mellitus in a Rodent Wounding Model

Wistar rodents with diabetes were actuated by a single intraperitoneal injection of STZ (50 mg/kg; Sigma-Aldrich, St. Louis, MO, USA) following our previous report [9,10,25]. Briefly, rodents with a glucose level more prominent than 300 mg/dl one week after injection were characterized as having effective acceptance of diabetes and were then utilized for subsequent experiments. To adjust the glucose level at 200 mg/dL, diabetic rodents were subcutaneously administered continual-acting insulin (1 to 2 unit/kg; Montards Novo Nordisk A/S, Bagsvaerd, Demark) until the animals were immolated. The wounding model was assessed a month after the STZ injection. The dorsum skin tissue of the Wistar rodents was excised to make a wounding area of 6 × 5 cm^2^. The whole skin was disfigured underneath the level of the dorsal fascia, and the edge of the wound defect was stitched set up with 4-0 silk stitches to forestall wound contracture. The wound was incidentally secured with lucent Tegaderm (3M HealthCare, Borken, Germany) till ESWT was initiated.

### 4.3. Experimental Design and Tissue Samples Collection

Eighteen 4-month-old male Wistar rodents (National Experimental Animals Production Center, Taipei, Taiwan) with STZ-prompted diabetes were separated into three groups (six rodents in each group): normal group, diabetic group, and ESWT group. The dorsal wounding model was made on all rodents. ESWT group was treated with two sessions of defocused shock waves (Reflector Type CP155; MTS, GmbH, Konstanz, Germany) utilizing 800 driving forces at 10 kV, proportionate to an energy flux density of 0.09 mJ/mm2 on days 3 and 7 after wounding following our former protocol [9,11]. Peri-wounding tissue tests were gathered at 3 and 10 days after ESWT treatment or those days in the normal control (NC) and control diabetes (DM) group. Immediately after excision, the skin tissue samples were snap-solidified in fluid nitrogen and put away until use.

### 4.4. Isoelectric Focusing, Gel Electrophoresis, Silver Staining, and Gel Imaging

Peri-wounding skin tissue protein was extricated utilizing PRO-PREPTM protein extraction solution (iNtRON Biotechnology, Gyeonggi-do, Korea) followed by a 2-D Clean-Up Kit (GE Healthcare Life Sciences, Uppsala, Sweden). Two-dimensional electrophoresis including isoelectric focusing and SDS-PAGE were executed on an Ettan IPGphor II/3 IEF system and SE 600 Ruby gel apparatus (GE Healthcare Life Sciences). Every sample was exposed to isoelectric focusing and SDS-PAGE in copy. The silver-recolored polyacrylamide gels were examined utilizing an ImageScanner (GE Healthcare Life Sciences). The gel pictures and spot patterns were coordinated and dissected utilizing Bio-Rad Proteoweaver 2-D Analysis Software ver. 4.0 (Bio-Rad Laboratories, Hercules, CA, USA).

### 4.5. Matrix-Assisted Laser Desorption Ionization Time-of-Flight (MALDI-TOF) Mass Spectrometry

The interesting spots were physically extracted, washed with deionized water, destained, rehydrated, reduced, and following trypsin digested. The extraction products were harvested with 1% trifluoroacetate in acetonitrile. Aliquots of the extracted digestion products were then stacked onto AnchorChip (Bruker Detection, Leipzig, Germany) trailed by MALDI-TOF evaluation utilizing an Ultraflex TOF/TOF mass spectrometer (Bruker Detection). The peptide mass information was submitted to the National Center for Biotechnology Information and Swiss-Port database utilizing Mascot (Matrix Science, Boston, MA, USA.) web search tools for peptide coordinating. The coordinated peptides that were considered as potential up-and-comers had the most noteworthy Mascot score (≥65) and a peptide sequence coverage of 20% of the coordinated peptide.

### 4.6. Immunohistochemical Staining

Specific proteins of proteome analyses were confirmed by IHC staining. Polyclonal antibodies against serine protease inhibitor (serpin) A3N (LSbio, Irvine, CA, USA) and Hemopexin (MyBioSource, San Diego, CA, USA) were utilized as the first antibodies. The slides were incubated with first antibodies (1:500 dilutions) for 1 h and afterward incubated with goat biotinylated anti-rabbit antibodies for another 30 min. Perception of the particular binding was developed by an enzymatic transformation of the chromogenic substrate 3,3′-diaminobenzidine into a brown precipitate by a horseradish peroxidase–diaminobenzidine staining kit (Thermo Systems, Minneapolis, MN, USA). In the wake of recoloring, the sections were mounted, cleared, cover-slipped, and inspected utilizing a Zeiss magnifying instrument (Zeiss, Gottingen, Germany).

### 4.7. Real-Time Quantitative Reverse Transcriptase-Polymerase Chain Reaction (RT-PCR) Analysis

All-out RNA was extracted from skin tissues utilizing TRIzol reagent (Invitrogen Life Technologies; Carlsbad, CA, USA). The 2 µg RNA contribution for cDNA synthesis was calculated by spectrophotometric optical density 260 estimation and cDNA was created with High Capacity cDNA Reverse Transcription Kit (Applied Biosystems, Foster City, CA, USA) depending on the manufacturer’s protocols. The expression of interesting genes was examined utilizing the TaqMan^®^ Gene Expression Assays bought from Applied Biosystems (Applied Biosystems, Foster City, CA, USA). The names of the analyzed gene, GenBank accession numbers, amplicon sizes, and assay ID of gene expression assays are list in Table 3. Expression of rat housekeeping genes, Actb (β-actin) was utilized for equalizing analyzed target genes expression in real-time quantitative RT-PCR. All reactions were completed in a 10 µL last volume containing 20 ng of cDNA, 5 µL of 2× TaqMan^®^ Universal PCR Master Mix, and 0.5 µL of 20× TaqMan^®^ Gene Expression Assay (Applied Biosystems). Real-time quantitative PCR was assessed using an ABI 7500 Fast Real-Time System (Applied Biosystems) and the thermal cycling programs were set as follows: 95 °C for 10 min followed by 40 repeats of PCR at 95 °C for 20 s and then 60 °C for another 1 min. The expression level of the gene of interest was equaled to the house-keeping control Actβ to calculate the relative threshold cycle (ΔCt) and the relative expression between two groups was determined by the comparative Ct (ΔΔCt) method [10].

### 4.8. Statistical Analysis

All qualities were presented as mean ± standard error. A matched t-test was utilized to distinguish the contrasts between the two groups of each protein level. The test was two-sided with statistical significance set at 0.05, and all calculations were made utilizing SPSS for Windows Release 13.0 software (SPSS, Chicago, IL, USA).

## Figures and Tables

**Table 1:** Mass spectrometric Features and Relative Expression of Emphatically Distinguished Protein in Skin Tissue of Diabetic Rodents That Had Gone Through Extracorporeal Shock Wave Therapy on Day 3 after Treatment.

							Relative Expression	
Spot	Identified Protein	Score	Molecular Weight (kilodalton)	Theoretical Isoelectric Point	Sequence Coverage (%)	Matched Peptides	in DM (Compared with NC)	in ESWT (Compared with DM)
1	Hemopexin	138	52.1	8.6	18.7	6	**↓**	****↑****
2	Alpha-2-HS-glycoprotein	112	38.8	6.1	15.6	4	****↓****	**↑**
3	Serine protease inhibitor A3N	270	46.8	5.2	22.2	8	**↑**	**↓**
4	Serine protease inhibitor A3N	335	46.8	5.2	29.9	9	**↑**	**↓**
5	Serine protease inhibitor A3N	442	46.8	5.2	28.2	13	**↑**	**↓**
6	Serine protease inhibitor A3N	419	46.8	5.2	26.8	9	**↑**	**↓**
7	Serine protease inhibitor A3N	304	46.8	5.2	27.0	10	**↑**	**↓**
8	Leukocyte elastase inhibitor A	245	42.9	5.9	24.0	11	**↑**	**↓**
9	Catechol O-methyltransferase	222	29.8	5.3	37.9	18	**↑**	**↓**

NC: normal control rats; DM: diabetic control rats; ESW2: diabetic rats received two sessions of extracorporeal shock wave therapy.

**Table 2:** Mass spectrometric Features and Relative Expression of Emphatically Distinguished Protein in Peri-wounding Tissue of Diabetic Rodents That Had Gone Through Extracorporeal Shock Wave Therapy on Day 10 after Treatment.

							Relative Expression	
Spot	Identified Protein	Score	Molecular Weight (kilodalton)	Theoretical Isoelectric Point	Sequence Coverage (%)	Matched Peptides	in DM (Compared with NC)	in ESWT (Compared with DM)
1	Serine protease inhibitor A3N	74	46.8	5.33	29	16	**↓**	**↑**
2	Tektin-4	69	52.7	58.3	34	17	**↓**	**↑**
3	Tropomyosin alpha-1 chain	71	32.7	4.69	33	10	**↓**	**↑**
4	Plectin-1	74	53.5	5.71	20	85	**↓**	**↑**
5	Lamin-A	53	74.6	6.54	25	13	**↓**	**↑**
6	Tropomyosin alpha-4 chain	147	28.5	4.66	50	15	**↓**	**↑**
7	Ferritin heavy chain	59	21.3	5.85	53	7	**↓**	**↑**
8	Myosin regulatory light chain 2, skeletal muscle isoform	102	19.1	4.82	58	11	**↓**	**↑**
9	Hemopexin	172	52.1	7.58	50	26	**↑**	**↓**

NC: normal control rats; DM: diabetic control rats; ESW2: diabetic rats received two sessions of extracorporeal shock wave therapy.

**Table 3:** TaqMan Gene Expression Assays for Real-Time Quantitative Reverse Transcriptase-Polymerase Chain Reaction Analysis of the Genes of the EGFR Pathway.

Gene	GenBank Accession No.	Amplicon Size (bp)	Assay Location	Assay ID (Applied Biosystems)
*Egfr*	NM_031507.1	104	241	Rn00580398_m1
*Kras*	NM_031515.3	88	448	Rn01463171_m1
*Raf1*	NM_012639.2	88	973	Rn00466507_m1
*Braf*	XM_231692.5	97	1843	Rn01500557_m1
*Mek1 (Map2k1)*	NM_031643.4	70	130	Rn00581264_m1
*Erk (Ephb1)*	NM_001104528.1	100	2611	Rn00557962_m1
*Elk3 (Kcnh8)*	NM_145095.1	87	1232	Rn00595436_m1
*Mekk1 (Map3k1)*	NM_053887.1	90	4577	Rn01490142_m1
*Jnkk (Map2k7)*	NM_001025425.1	104	122	Rn01403106_m1
*Jnk (Mapk8)*	XM_341399.5	85	247	Rn01218952_m1
*Jun*	NM_021835.3	106	1644	Rn99999045_s1
*Actb*	NM_031144.2	884	91	Rn00667869-m1

## Abbreviations

DM	Diabetes Mellitus; in this study mean “control diabetes”
EGFR	Epidermal growth factor receptor
ESWT	Extracorporeal shock wave therapy
HRP-DAB	Horseradish peroxidase-diaminobenzidine
IHC	Immunohistochemical
MALDI-TOF	Matrix-Assisted Laser Desorption Ionization Time-of-Flight
NC	Normal control
Serpin	Serine protease inhibitor
STZ	Streptozotocin
